# Iterative consensus spectral clustering improves detection of subject and group level brain functional modules

**DOI:** 10.1038/s41598-020-63552-0

**Published:** 2020-05-05

**Authors:** Sukrit Gupta, Jagath C. Rajapakse

**Affiliations:** School of Computer Science and Engineering, Nanyang Technological University, Singapore, Singapore

**Keywords:** Network models, Neural circuits

## Abstract

Specialized processing in the brain is performed by multiple groups of brain regions organized as functional modules. Although, *in vivo* studies of brain functional modules involve multiple functional Magnetic Resonance Imaging (fMRI) scans, the methods used to derive functional modules from functional networks of the brain ignore individual differences in the functional architecture and use incomplete functional connectivity information. To correct this, we propose an Iterative Consensus Spectral Clustering (ICSC) algorithm that detects the most representative modules from individual dense weighted connectivity matrices derived from multiple scans. The ICSC algorithm derives group-level modules from modules of multiple individuals by iteratively minimizing the consensus-cost between the two. We demonstrate that the ICSC algorithm can be used to derive biologically plausible group-level (for multiple subjects) and subject-level (for multiple subject scans) brain modules, using resting-state fMRI scans of 589 subjects from the Human Connectome Project. We employed a multipronged strategy to show the validity of the modularizations obtained from the ICSC algorithm. We show a heterogeneous variability in the modular structure across subjects where modules involved in visual and motor processing were highly stable across subjects. Conversely, we found a lower variability across scans of the same subject. The performance of our algorithm was compared with existing functional brain modularization methods and we show that our method detects group-level modules that are more representative of the modules of multiple individuals. Finally, the experiments on synthetic images quantitatively demonstrate that the ICSC algorithm detects group-level and subject-level modules accurately under varied conditions. Therefore, besides identifying functional modules for a population of subjects, the proposed method can be used for applications in personalized neuroscience. The ICSC implementation is available at https://github.com/SCSE-Biomedical-Computing-Group/ICSC.

## Introduction

The human brain consists of spatially distributed but functionally linked regions that give rise to its different behavioral and cognitive capabilities. The organization of brain functional networks, as modeled by fMRI data, can be studied at various scales, ranging from nodal or edge properties at microscale, to properties such as path length at macroscale. Recently, mesoscale properties of brain functional networks have been successfully investigated by decomposing them into several modules (for review see^1^). Distinct functional modules have been shown to be representatives of different systems performing specialized neurophysiological functions in the brain^2,3^. The detection of these modules affects the identification and classification of nodes as hubs coordinating inter-modular or intra-modular coordination^4^. These hub regions have been shown to have increased susceptibility in case of brain disorders and cause major functional disruption in case of brain injury^5^. Additionally, changes in modular structure have been reported in neurological ailments like Alzheimer disease^6^, schizophernia^7^, post traumatic stress disorder^8^ and chronic pain^9^. These factors together make the reliable detection of brain modules an important factor in understanding the dynamics between brain functional architecture and brain disease. This has prompted several investigators to find methods that identify functional modules of the brain.

In this study, we detect brain functional modules from the connectivity matrix obtained from fMRI BOLD time-series signal of functionally diverse brain regions. Previous studies in the area^2,10–12^, have made an assumption that the modules obtained from applying module detection algorithms on an averaged connectivity matrix from multiple subjects reliably represent the modular structure of underlying subjects. This approach is flawed because averaging of subject connectivity matrices leads to the loss of important subject-level features^13–15^ and ignores the heterogeneous inter-subject differences in brain functional architecture^16^ that are consequential while detecting group-level brain functional modules. The variability in functional architecture across individuals may not be limited to variation in the function of brain regions but also grouping/sub-division of functional modules in individuals. Therefore, detecting group-level modules from multiple subjects in the ensemble should not only consider modular architecture for all the subjects but also allow for the variablility in their modular structure.

We hypothesize that the most representative group-level modularization is maximally similar to the modularization of the individuals (note that these are different from the subject-level modularizations and these correspond to the modularizations for scans or subjects, as the case maybe. We refer to these as *individual-level modularizations*). Based on this hypothesis, we develop a new approach called *iterative consensus spectral clustering* (ICSC) based on consensus clustering^17^ and multiclass spectral clustering^18^. We define the *consensus-cost* based on similarity between group-level and individual-level modularizations and iteratively refine both the individual-level and group-level modules making them more similar to each other. This procedure gives a reliable group-level representative modular structure by using the group-level modular information as a prior to refine individual-level modules. We allow individual-level modularization variability by not only variable modular structure but also varied number of modules at individual-level. Recent studies have shown that weak edges predict differences in fluid intelligence^19,20^ and differentiate people suffering from schizophrenia from healthy controls^21^, which necessitates that loss of information from steps like thresholding and binarizing should be avoided. We avoid loss of weak functional connections and connection weights by considering unthresholded (dense), weighted individual-level functional connectivity matrices. We show that the ICSC algorithm can compute biologically plausible modules without setting any hard conditions for specific modules to be detected (as in^12^) or varying parameters like network thresholds or resolution limit. The ICSC algorithm found several smaller modules that were not detected by previous modularizations.

Using resting-state fMRI scans obtained from 589 subjects from the Human Connectome Project (HCP), we use ICSC algorithm to detect group-level modules from multiple subjects and subject-level modules from multiple scans of the subject. On computing variability using the group-level and subject-level modules, we found lower intra-subject variability in comparison with inter-subject variability. The brain regions involved in elementary tasks like vision and motor were found to be stable both across subjects and across scans. We compared the modularization obtained from the ICSC algorithm with previous methods such as the Louvain^22^, InfoMap^23^, and Asymptotical Surprise^10^ and found that these algorithms give a few large, functionally relevant set of modules, whereas the ICSC algorithm gives smaller biologically plausible modules, which are most similar to individual-level modularizations. Furthermore, we found that the iterative procedure in the ICSC algorithm, gives significantly more representative subject-level modules by using multiple subject scans from the HCP dataset. For a quantitative comparison, we generated synthetic networks having similar modular structure to resting-state functional connectivity networks obtained from human fMRI scans; and the ICSC algorithm was able to detect group-level modules stably across different noise levels and nodal variations.

## Methods

Given a set of resting-state fMRI brain scans, we first derive functional connectivity matrices of the brain networks. For $k$th individual, the functional network ${G}^{k}=(\Omega ,{{\bf{W}}}^{k})$ where $\Omega$ denotes the set of functionally relevant regions of interests (ROI) or nodes of the network and ${{\bf{W}}}^{k}$ denotes functional connectivity matrix derived from the fMRI scan.

### Module detection at individual-level

A functional modularization (i.e., detecting the functional modules given the brain functional connectivity matrix) is a partitioning of $\Omega$ into disjoint sets or modules such that the connectivity among the nodes within a module is higher than the connectivity between the nodes of different modules. Given the functional brain network ${G}^{k}$ for $k$th individual, a modularization finds a vector ${{\boldsymbol{s}}}^{k}=\{{s}_{i}^{k}{\}}_{i\in \Omega }$ where each element ${s}_{i}^{k}$ corresponds to a module label assigned from a set $\{1,2,\ldots ,{L}^{k}\}$ and ${L}^{k}$ denotes the number of modules.

The challenge in finding individual-level functional modules is two folds. First, the number of modules ${L}^{k}$ of an individual $k$ is unknown. Second, the partitioning of the nodes at individual level, ${{\boldsymbol{s}}}^{k}$, should be consistent with the consensus modules at the group-level. Given the number of modules ${L}^{k}$, we denote assigning a modularization ${{\boldsymbol{s}}}^{k}$ for the subject $k$ as

1 $${{\boldsymbol{s}}}^{k}\leftarrow {{\boldsymbol{s}}}^{k}({L}^{k})$$

where ${s}^{k}({L}^{k})$ denotes the process of partition the set Ω of brain ROI into ${L}^{k}$ modules.

### Spectral clustering on normalized cut cost

Modules from a network are derived by optimization of the quality function such as the Newman’s modularity^24^, the Code length^23^ or the normalized cut cost^25^. Previous studies have shown an existence of a resolution bias for the modularization quality functions like Code length, Newman’s modularity and the normalized cut cost function, whereby these quality functions have an inherent inability towards detecting very small or large modules. This resolution bias has been shown to severely curtail the detection of small albeit functionally relevant brain modules^11^. However, it was pointed out that although these quality functions suffer from different resolution biases, they gave similar results when forced to converge to a specific number of modules^26^. Unlike the normalized cut-cost, the Code length and Newman’s modularity objective functions cannot be forced to detect a fixed number of modules, they can be run multiple times to determine suitable parameters values that lead to convergence to the suitable number of modules. This is an important distinction between the objective functions for our case, because using prior neuroscientific knowledge, we can get a range of the number of functional modules that are present across subjects. Additionally, unthresholded resting-state functional connectivity networks worsen the module detection capability of Code length and Newman’s modularity. On the other hand, multiclass spectral clustering^18^ used to optimize the normalized cut-cost objective function solves the problem in eigenspace of the network Laplacian and is therefore not affected by the density of networks.

A functional modularization ***S***^*k*^ is also represented by a set of binary matrices as ${{\boldsymbol{X}}}^{k}={\{{{\boldsymbol{x}}}_{l}^{k}\}}_{l\mathrm{=1}}^{{L}_{k}}$ where $L$ th module ${{\bf{x}}}_{l}^{k}={({x}_{li}^{k})}_{i\in \Omega }$ such that ${x}_{li}^{k}=1({s}_{i}^{k}=l)$ and $\mathrm{1(}\,\cdot \,)$ denotes the identity function. The degree matrix ${{\boldsymbol{D}}}^{k}$ of the connectivity matrix ${{\bf{W}}}^{k}$ defined as the diagonal matrix having diagonal elements as the degree of the corresponding nodes is given by

2 $${{\boldsymbol{D}}}^{k}={\rm{diag}}({{\boldsymbol{W}}}^{k}{{\bf{1}}}_{|\Omega |})$$

where ${{\bf{1}}}_{|\Omega |}$ is a vector of ones containing $|\Omega |$ elements.

The *normalized cut-cost* objective function is given by:

3 $${\rm{cut}}-{\rm{cost}}({{\boldsymbol{X}}}^{k})=\frac{1}{{L}^{k}}\mathop{\sum }\limits_{l\mathrm{=1}}^{{L}^{k}}\left(1-\frac{{({{\boldsymbol{x}}}_{l}^{k})}^{T}{{\boldsymbol{W}}}^{k}{{\boldsymbol{x}}}_{l}^{k}}{{({{\boldsymbol{x}}}_{l}^{k})}^{T}{{\boldsymbol{D}}}^{k}{{\boldsymbol{x}}}_{l}^{k}}\right)$$

The minimization of the cut-cost in (3) is performed by multiclass spectral clustering (SC)^18^. This method gives an optimal solution and a modularization of *G*^*k*^. Multiclass spectral clustering is fast and more robust than random initialization. Given the functional connectivity *G*^*k*^ and the number of modules *L*^*k*^, we find the modularization denoted in (1) by using spectral clustering.

### Group-level consensus modules

Earlier works have mostly focused on modular detection at the group-level^2,10,11^ as a representation of the modules of the individuals. Such approaches perform module detection on an averaged connectivity matrix ignoring the variability in functional architecture of individuals^14,27,28^. We propose to use a consensus clustering approach^17^ since it considers the individual-level modularizations while deriving a group consensus matrix. The individual-level modular matrix is a binary matrix where an element equals one if the corresponding nodes belong to the same modules and is zero, otherwise. For the $k$th individual, the individual-level modular matrix is given as ${{\boldsymbol{\Pi }}}^{k}=\{{\pi }_{ij}^{k}|{\pi }_{ij}^{k}\mathrm{=1}({s}_{i}^{k}={s}_{j}^{k}{)\}}_{i,j\in \Omega }$. The *group consensus matrix* ${\boldsymbol{\Pi }}$ is computed by adding the individual-level modular matrices for all the individuals:

4 $${\boldsymbol{\Pi }}=\sum _{k}{{\boldsymbol{\Pi }}}^{k}$$

The group consensus matrix is a multilevel affinity matrix and is used to find the group-level modularization. By using the spectral clustering on ${\boldsymbol{\Pi }}$ and assuming an $L$ number of modules, we denote a group-level modularization as:

5 $${\boldsymbol{s}}\leftarrow {\boldsymbol{s}}(L)$$

### Iterative consensus spectral clustering (ICSC)

Iterative Consensus Spectral Clustering (ICSC) extends the multiclass spectral clustering to attain an individual-level modularizations close to the group-level consensus modularization. The ICSC iteratively maximizes the *consensus-cost* or minimizes the mean distance (similarity) between the group-level consensus modularization and the individual-level modularizations. We hypothesize that the individuals share common features in their functional architecture such as the number of modules, which can be inferred from the group level features, whereas in case of multiple scans from the same subject, the functional architecture across the scans is same but is distorted due to scanner artefacts or mind wandering during acquisition.^16,29–31^.

The consensus cost of individual-level modularizations $\{{{\boldsymbol{s}}}^{k}\}$ and group-level modularization ${\boldsymbol{s}}$ is given by

6 $${\rm{consensus}}-{\rm{cost}}({\boldsymbol{s}},\{{{\boldsymbol{s}}}^{k}\})=\mathop{\sum }\limits_{k\mathrm{=1}}^{K}\,{\rm{sim}}({\boldsymbol{s}},{{\boldsymbol{s}}}^{k})$$

where ${\rm{sim}}$ measures the similarity between two modularizations and $K$ denotes the number of individuals.

We use the symmetric measure Adjusted Mutual Information (AMI)^32^ to measure the similarity between the group-level and an individual-level modularization. The AMI is an extension to Mutual Information (MI) score and accounts for the chance. We avoid using MI and normalized MI (NMI) since they increase for two partitions with high number of modules, irrespective of the information shared^33^. Similarly, we also avoid using Adjusted Rand Index since it is preferred for modularizations with large similarly sized modules^34^, whereas brain modules are non-uniformly sized^35^. The AMI between two modularizations ${\boldsymbol{s}}$ and ${{\boldsymbol{s}}}^{k}$ is given by:

7 $${\rm{sim}}({\boldsymbol{s}},{{\boldsymbol{s}}}^{k})=\frac{MI({\boldsymbol{s}},{{\boldsymbol{s}}}^{k})-{\rm{mean}}(MI({\boldsymbol{s}},{{\boldsymbol{s}}}^{k}))}{{\rm{\max }}(H({\boldsymbol{s}}),H({{\boldsymbol{s}}}^{k}))-{\rm{mean}}(MI({\boldsymbol{s}},{{\boldsymbol{s}}}^{k}))}$$

where $H$ is the entropy of a partition and $MI$ is the mutual information between the two partitions.

The ICSC optimizes the consensus-cost by alternatively (i) finding the number of modules ${L}^{k}$ and $L$ at the individual-level and group-level, respectively, and (ii) minimizing the normalized cut-cost(${{\bf{X}}}^{k}$) to attain individual-level modularizations. Finding the optimal number of clusters/modules for a given data is an open problem and there are various module quality indices available which aid in selecting a reasonable number of modules (see^36^ for a review). For our data, we found that there was no agreement on the optimal number of modules suggested by different module quality indices. We, therefore, decided to approach the problem in terms of the information content of the group consensus matrix ${\boldsymbol{\Pi }}$.

Given that there exists community structure in ${\boldsymbol{\Pi }}$, then $L < |\Omega |$, which points to presence of redundant information in ${\boldsymbol{\Pi }}$. For ${\boldsymbol{\Pi }}$, the redundancy can be visualized as shared information between ROI which are part of the same modules across many individual-level modularizations which makes them linearly-dependent. Thus finding the number $L$ of group-level modules can be visualized as the problem of finding the optimal number of dimensions that can balance the trade-off between independence and redundance, minimizing the redundance. This can be solved using the formulation given in^37^. We transform the ROI in the eigenspace, where the orthogonal eigenvectors are considered to represent modules. However, not all eigenvector directions contribute equally and the important eigenvectors can be computed from their eigenvalues. We estimate the number of dimensions by finding the “elbow” of the scree plot of the group consensus matrix, ${\boldsymbol{\Pi }}$:

8 $$L={\rm{elbow}}({\boldsymbol{\Pi }})$$

where the ${\rm{elbow}}$ function computes the point of maximum curvature of eigenvalues^38^ in the range ${L}_{{\rm{\min }}}$ and ${L}_{{\rm{\max }}}$. The number of individual-level modules, ${L}^{k}$, are found by maximizing the similarity between the group-level ${\boldsymbol{s}}$ and individual-level modularizations, $\{{{\boldsymbol{s}}}^{k}\}$, as

9 $${L}^{k}=\mathop{{\rm{argmax}}}\limits_{l}\,{\rm{sim}}({\boldsymbol{s}},{{\boldsymbol{s}}}^{k}(l))$$

The minimum number of modules, ${L}_{{\rm{\min }}}$, can be fixed on prior studies in the area. However, the choice for the maximum number of modules, ${L}_{{\rm{\max }}}$, has a tradeoff in terms of the time taken for convergence and the choices available for $l$ in Eq. 9 for maximization of consensus-cost. Lower than optimal values of ${L}_{{\rm{\max }}}$ will have a sharp drop-off near ${L}_{{\rm{\max }}}$ in the histogram of the distribution of number of individual-level modules, ${\{{L}^{k}\}}_{k\mathrm{=1}}^{K}$. The sharp drop-off corresponds to individual-level modularizations that were assigned sub-optimal number of modules, ${L}^{k}$, which led to lower value for the consensus-cost.

The ICSC begins by performing individual-level modularization using SC and uses them to derive a group-level modularization. Thereafter, it iteratively finds the optimal numbers of modules $L$ and ${L}^{k}$ at the group-level and individual-level, respectively. First, it finds the individual-level modules ${{\boldsymbol{s}}}^{k}$ and then uses them to derive the group-level consensus matrix and finds the optimal number of group-level modules $L$, and so on until convergence. SC is used to obtain modularization at the individual-level and group-level, and the algorithm converges when no change in the *consensus-cost* is observed. In order to initialize $\{{{\boldsymbol{s}}}^{k}\}$ and ${\boldsymbol{s}}$, we compute the most suitable initial ${L}^{k}$ and $L$ using the elbow function with ${{\boldsymbol{W}}}^{k}$ and ${\boldsymbol{\Pi }}$, respectively. The Algorithm 1 gives the complete ICSC procedure.

### Inter-subject variability of functional modules

Several neuroimaging studies have shown pronounced inter-subject differences in the brain functional architecture distributed non-uniformly across the cortex^16,39,40^. Functional variability across subjects is a result of the combined effect of genetic and environmental factors that impact functional systems differently across subjects^41^. The variability of modular membership of brain regions across subject helps understand individual differences in cognition and behavior^15^.

Using individual-level ${{\boldsymbol{s}}}^{k}$ and group-level modularizations ${\boldsymbol{s}}$ from the ICSC algorithm, the nodes with high variability in their modular assignment over individuals can be identified. For this, we first match the labels in the group-level and individual-level modularizations by using a Jaccard Index matching scheme described in^17^ and then compute the metric *node purity* for node $i\in \varOmega$ as

10 $${p}_{i}=\frac{1}{K}\sum _{k}1({s}_{i}^{k}={s}_{i}^{\ast })$$

**Algorithm 1:**
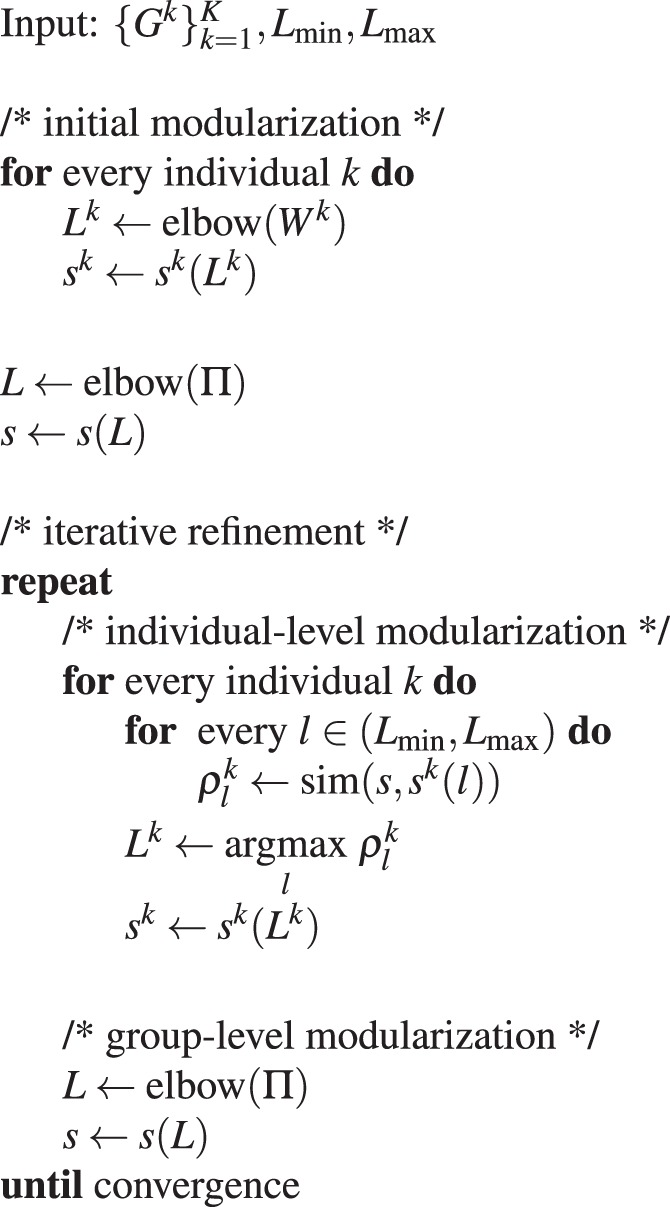
Iterative consensus spectral clustering.

where ${s}_{i}^{\ast }$ is the most frequently assigned module for node $i$ across the individuals. We derive the purity of a module by averaging the purities of nodes belonging to the module. The module purity gives an idea about the variability of a module across the individuals.

### Detecting subject-level functional modules

Although group-level features render properties that are common to a group of individuals, finding neuro-functional features at the subject-level has significant applications in personalized neuroscience. Finding accurate modularization at subject-level is important to pinpoint susceptibility of the subject to diseases^7,42,43^, and detect different brain states^44^ and anatomical locations of the hubs in the subject’s brain networks^5,10^. However, it is also important to know if brain functional modules are reproducible across multiple scans of the same subject.

Previous studies show that there exist significant differences across scans of even the same subject^16,29^. The inter-scan variability of the functional modules of the same individual arises due to noise in MRI acquisition and different functional activities during resting-state sessions^31^. The functions of brain regions (and therefore, functional modules) of a subject do not change much from one scan to the other and consequently the functional modules should have similar stability across scans.

We need a modularization method that considers each scan-level modular structure ${{\boldsymbol{s}}}^{k\sigma }$ and uses it to derive the subject-level modularization ${{\boldsymbol{s}}}^{k}$, where $\sigma$ denotes a particular scan of the subject $k$. We propose the use of ICSC algorithm to derive a representative subject-level modularization from multiple scan-level modularizations. A good modularization algorithm should give less variability for intra-subject functional modules compared to the inter-subject variability of functional modules. We used the data from the HCP which involves multiple resting-states scans from the same subject. To calculate intra-subject variability, we obtain the purity ${p}_{i}^{k}$ of node $i$ across multiple scans $\sigma$ of the subject $k$ similar to (10). Then, for multiple subjects, we obtain the purity ${p}_{i}$ of the node as ${p}_{i}=\frac{1}{K}{\sum }_{k}{p}_{i}^{k}$

## Results

### Dataset

The primary data used in this study is from the Human Connectome Project (HCP) of the Washington University-Minnesota Consortium^45,46^ openly available at https://www.humanconnectome.org. The data was collected on a Siemens Skyra 3T scanner and included state-of-the-art whole-brain MRI acquisition with structural, functional, and diffusion-weighted imaging. Functional scans were acquired in two sessions with each session consisting of two 15 minutes resting-state run in different phase-encoding directions (left-right and right-left). We analyzed the scans of subjects who completed all four resting-state scan runs (two sessions, each with two encoding runs), which resulted in $n$ = 589 subjects (males = 278, mean age = 28.33 years, range = 22 to 37 years). The HCP minimal processing pipeline^46,47^ was used for preprocessing, which included projection to the surface space, FWHM smoothing (2 mm), ICA + FIX denoising with minimal high-pass filtering and MSMall surface registration.

### Brain functional ROI

As functionally relevant regions of interest (ROI), we used the 264 brain regions identified by Power *et al*.^2^. The Power atlas contains both anatomically and functionally diverse brain regions and defines the functional role played by each ROI. We calculated the mean time series of all voxels within a sphere of radius 2.5 mm of each ROI. The Pearson correlation was used to compute the functional correlation between two ROI. We considered all the positive correlations between the ROIs, covering the entire cerebral cortex.

### Group-level modules from multiple subject data

For each subject, the functional connectivity matrix was derived from the Pearson correlation of time-series between ROIs from each scan. The connectivity matrices from four scans were averaged to obtain the connectivity matrix for the subject. Power *et al*. found 14 modules and Yeo *et al* found 18 modules, and we assume that there are at least 5 functional systems discernible in the resting-state fMRI scans viz. sensory somatomotor, visual, default mode, cingulo-opercular task control, and fronto-parietal task control. We, therefore, fixed the minimum number of modules ${L}_{{\rm{\min }}}=5$ and started with ${L}_{{\rm{\max }}}=20$ for the ICSC algorithm. We increased ${L}_{{\rm{\max }}}$ in steps of 5 and initiated 10 independent runs for each value. We observed a sharp drop in the histogram of $\{{L}^{k}\}$ distribution for ${L}_{{\rm{\max }}}=\mathrm{\{20,25\}}$ and observed that for ${L}_{{\rm{\max }}}=\mathrm{\{30,35,40\}}$ the histogram for $\{{L}^{k}\}$ was smooth. We also observed optimal consensus-cost at ${L}_{{\rm{\max }}}\mathrm{=30}$ and therefore selected this value for the rest of the experiments. Refer to Supplementary Materials for more details.

**Figure 1:**
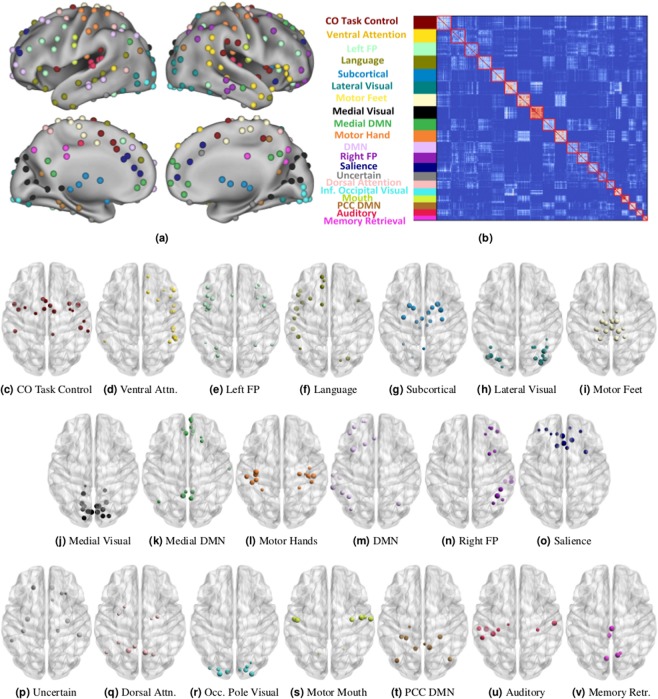
The twenty group-level modules detected by the ICSC algorithm on the resting-state fMRI scans from the HCP dataset. (**a**) The functional ROI colored according to the modules they belong to. (**b**) the group consensus matrix with reordered node indices to bring nodes in the same module together. **(c**) to (**v**) plotted using BrainNetViewer^54^ show the ROIs belonging to each module ordered in the descending order of module size. The modules are given names based on functional networks identified by earlier studies or on the anatomical location of constituent regions. (**a**,**b**) have been obtained from Gupta *et al*.^55^ with the permission of the authors.

We ran 100 independent runs of the ICSC algorithm until convergence on both the datasets. The number of iterations the ICSC procedure took to converge ranged from 4 to 19 (average $8.6\pm 3.5$) for the HCP dataset. For the results of the algorithm to be reliable, the final group-level modules detected from the independent runs should be identical. We found that the AMI between the group-level modularizations of different runs was $0.84\pm 0.08$ initially and $0.94\pm 0.02$ at the convergence, which shows that the results are highly reproducible. The ICSC algorithm detected 20 group-level modules with the sizes of modules ranging from 6 to 18 (Fig. 1). Out of the 100 independent runs, we selected the run with the maximum consensus-cost and study the individual-level and group-level modularizations for this run.

The group-level modularization found by the ICSC algorithm corresponds to previously defined functional networks of the human resting-state brain^2,48–50^. We recovered several sub-modules from previously detected modules. We recovered the cingulo-opercular network containing regions from anterior cingulate areas and supplementary motor area (Fig. 1(c)). The frontoparietal task control network was split into two subnetworks, left and right fronto-parietal task control networks (Fig. 1(e,n). The ventral and dorsal attention networks were also found with the ventral attention network having regions from right superior temporal gyrus, right inferior and superior frontal gyrus (Fig. 1(d)) and the dorsal attention network containing regions from the superior parietal lobe and superior precentral gyrus (Fig. 1(q)). The default mode network (DMN) was split into three modules, a module with regions from the anterior and posterior cingulate (Fig. 1(k)), a module with regions in the posterior cingulate cortex (PCC) and precunius area (Fig. 1(t)) and a module with regions in the dorso-lateral frontal, left middle temporal and lateral inferior parietal area 0(m)). The division of default mode network along medial and temporal regions is consistent with the prior literature^51^. The visual system was split into three modules, each representing a set of regions from medial (Fig. 1(j)), lateral (Fig. 1(h)) visual cortex and the occipital pole (Fig. 1(r)), which is consistent with intensive anatomical differentiation of the visual cortex^52^. The medial and lateral visual modules are known to be involved in semantic language processing whereas occipital pole visual module is involved in space perception^53^. We also detected a language module containing regions from the left middle temporal and inferior frontal gyrus, dorsolateral superior frontal and cerebeullum (Fig. 1(f)). The sensory/somatomotor network was divided into different areas for feet (Fig. 1(i)), hands (Fig. 1(l)) and the mouth (Fig. 1(s)) with regions lying in the precentral to postcentral gyrus. A module representing the memory retrieval system containing posterior medial regions from the cingulate gyrus and precuneus (Fig. 1(v)) was also found. Finally. a module containing inferior regions from the temporal and frontal lobe (Fig. 1(p)) was detected, whose function is unknown. We detected an independent module corresponding to the auditory system containing regions from superior temporal gyrus (Fig. 1(u)). We also recovered a module containing subcortical regions from the cerebellum, thalamus, amygdala, and basal ganglia (Fig. 1(g)). A module with regions from the middle frontal gyrus and anterior cingulate gyrus that form the salience network (Fig. 1(o)) was also detected.

We found considerable variation in the number of individual-level modules at convergence (mean = $20.91\pm 2.76$), which needs to be taken into account while detecting group-level modular structure. Since many individual-level modularizations have greater or fewer number of modules than the group-level modules, we further studied which modules were different in individual-level modularizations. We found that the subcortical, language and ventral attention modules were subdivided into smaller modules for modularizations with high number of modules, whereas the PCC DMN, motor hand and feet, right fronto-parietal task control and salience modules were combined with other modules for modularizations with lesser number of modules.

In order to understand the reproducibilty of ICSC modularization on a subset of the dataset, we divided the HCP dataset into three folds, such that the average age of the subjects and the gender ratio in each fold is maintained. We ran 20 independent runs on the subject data in each fold and selected the run with the maximum consensus-cost. We found that the modularizations obtained from different folds were not only similar to each other (AMI = $0.87\pm 0.0003$), but also to the one obtained with the whole dataset (AMI = $0.92\pm 0.01$). The corresponding results with multiple folds are given in the supplementary materials.

### Validation on an independent dataset

In order to further validate ICSC algorithm, a dataset of 116 subjects provided by the Creativity and Affective Neuroscience Lab, Brain Imaging Center of Southwest University (http://www.qiujlab.com). The dataset contained rs-fMRI scans collected from 3T MRI scanners for an 8-min period for 116 cognitively normal subjects with an average age of $23.54\pm 2.97$ with 44 male participants at Xinan (First Affiliated Hospital of Chongqing Medical School in Chongqing, China). We excluded the participants with age greater than 35 (to make it consistent with the HCP dataset). The preprocessing was performed using DPARSF^56^ (http://restfMRI.net), which is a toolbox based on the SPM8 software package. They discarded the first 10 EPI scans and the remaining scans underwent slice timing correction, motion correction, spatial normalization to standard Montreal Neurological Institute (MNI) space followed by spatial smoothing. To remove spurious correlations band-pass temporal filtering (0.01–0.1 Hz), nuisance signal removal from the ventricles and deep white matter and regression of 24 head motion parameters was applied. They also performed scrubbing movement correction Power *et al*.^57^ and removed subjects with mean framewise displacement greater than 10%. For more details on preprocessing please refer to Cheng *et al*.^58^.

We ran 100 independent runs of the ICSC algorithm on the validation dataset. The number of iterations the ICSC procedure took to converge ranged from 2 to 32 (average $9.3\pm 5.5$). We selected the run with the maximum consensus-cost and studied the group-level modularizations for this run. For the validation dataset, the ICSC algorithm detected 20 group-level modules with the sizes of modules ranging from 7 to 19. Although there are differences in the participant demography, scanning protocol, and data preprocessing steps, we obtained a similar modularization as with the HCP dataset (Figures in Supplementary Materials). We were able to detect sub-modules of the default mode network, the motor network, the visual network similar to the HCP dataset.

### Inter-subject variability of functional modules

**Figure 2:**
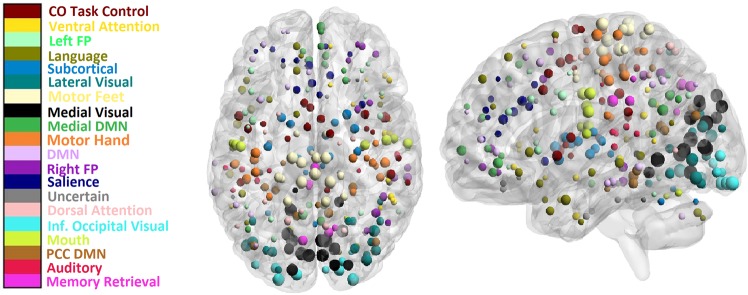
The inter-subject purity corresponding to different anatomical locations of the ROI (bigger size points to higher purity) along with their functional membership denoted by the ROI color.

**Figure 3:**
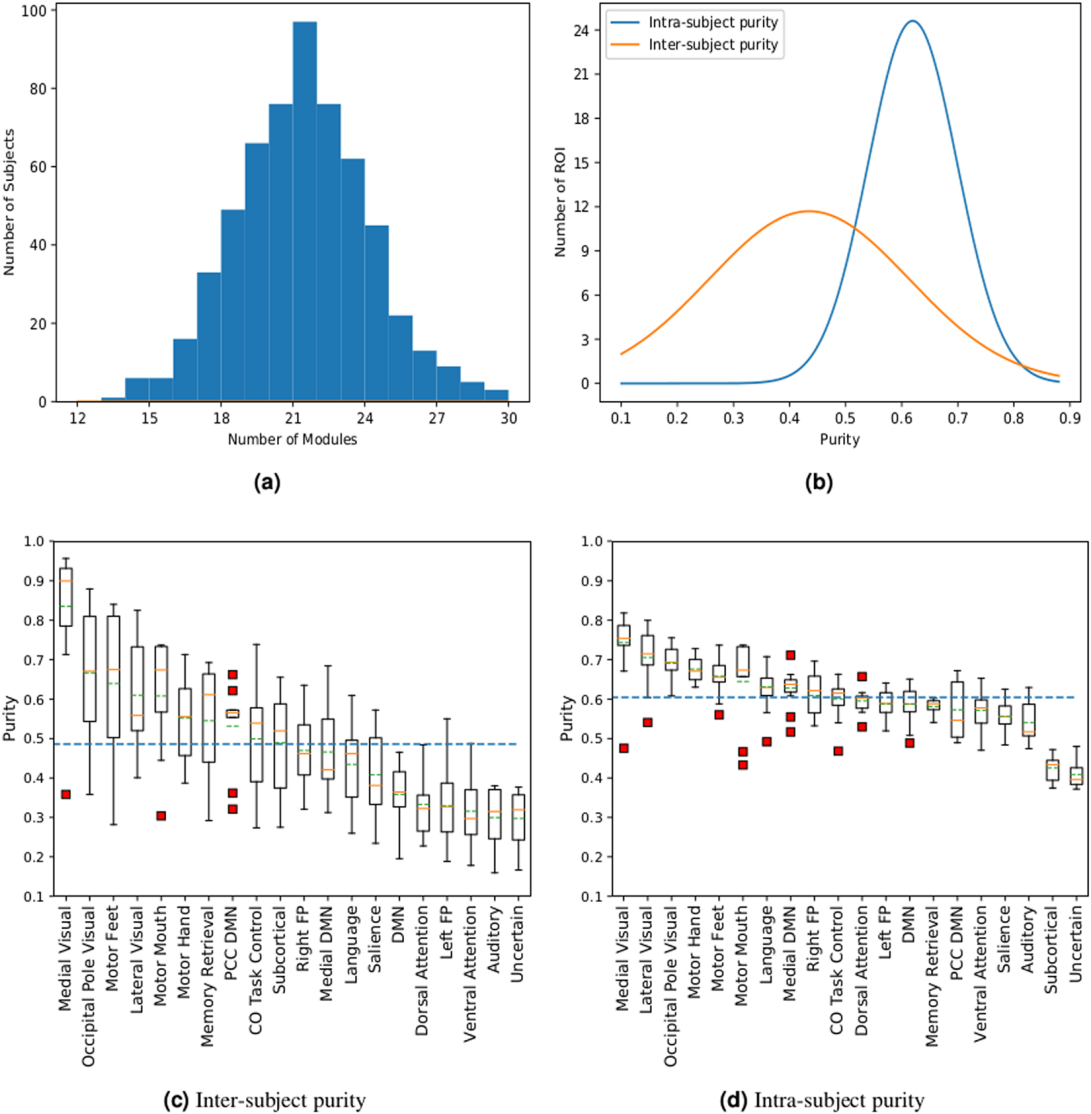
Analysis of inter-subject and intra-subject variability of the ROI of the functions modules of the human brain. (**a**) shows the distribution of modules across subjects for group-level modularization. (**b**) shows the distribution of the purities of different ROI in the brain for inter-subject and intra-subject variability. (**c**,**d**) show the box plot for the purities of ROIs of different modules (green dotted line in the boxes shows the average purity for the module, blue dotted line shows the average for all the ROIs) for inter-subject and intra-subject variability, respectively.

To study the inter-subject variability of brain functional modules, we computed nodal purities ${p}_{i}$ using modularizations obtained from the ICSC algorithm. We found that nodal purity had a wide distribution (average $0.483\pm 0.184$, Fig. 3(b)), showing that while some ROI are involved in the same function across individuals, others are involved in varied individual-specific tasks. This is consistent with prior findings that inter-subject variability is non-uniformly distributed across the brain^27^. We found the regions in the occipital lobe, anterior/posterior central paracentral lobe have the highest purity while regions in the lateral frontal, subcortical, and anterior/post central cigulate gyrus have low purity (Fig. 2). This is consistent from a brain evolutionary standpoint that the phylogenetically late developing frontal, temporal, and parietal association cortex areas involved in higher cognitive functions like reasoning lead to individual variability whereas the regions in the motor and visual cortex are consistent across individuals^59^.

From a functional perspective, we observed that the ROI classified in the medial visual, occipital visual, and somatomotor hand modules had a consistently high purity whereas the ROIs in the fronto parietal, cingulo opercular and occipital default mode had high variation across individuals (Fig. 3(c)). The low variability of visual and motor systems has been attributed to the high local information processing whereas modules like fronto-parietal, cingulo-opercular, and deafult mode are consistently involved in information processing between different functional systems^60^. We have provided the purity of ROI along with the anatomical locations in the Supplementary material. The wide distribution in the purity of modules when studying inter-subject differences is consistent with previous findings on the variability in brain functional systems^16^.

### Subject-level modules from multiple scans

To detect subject-level modules, we used multiple scans of the same subject from the HCP and computed the consensus modularization by using the ICSC algorithm. In this case, the individual modularizations, ${{\bf{s}}}^{k\sigma }$, represent scan-level modules whereas the group-level modularization, ${{\bf{s}}}^{k}$, represents subject-level modules. It took 1 to 4 ($1.58\pm 0.7$) iterations for the ICSC algorithm to converge for each subject in the HCP. We computed the similarity (using AMI) between scan-level modularizations of the same subject and between the scan-level and subject-level modularizations. The increase in similarity between scan-level modularizations at the start and convergence of ICSC algorithm was statistically significant (with a paired t-test p-value ${\mathrm{ < 10}}^{-51}$). Similarly, the increase in similarity between scan and subject-level modularizations at the start and convergence of ICSC algorithm was also statistically significant (with a paired t-test p-value ${\mathrm{ < 10}}^{-45}$). This shows that ICSC algorithm renders similar scan-level modularizations and a more representative subject-level modularization as a consensus among the scan-level modularizations.

For intra-subject variability of modules, we expected to see a high and similar purity values for the ROI since there is no variation of the regional functions across scans of the same subject. Correspondingly, we found a narrow spread of values for node purity (average $0.61\pm 0.09$ as seen in Fig. 3(b)). The spread of purity is higher for inter-subject variability than intra-subject variability. Intra-subject variability can be attributed to the noise artifacts in fMRI data and since ROI in the inferior temporal gyrus, inferior frontal gyrus and subcortical regions are known to have the highest noise confounds^16,29^, they should therefore have the lowest purity. Correspondingly, we found the subcortical and uncertain modules that contain nodes from these regions to have the lowest purity (Fig. 3(d)).

### Comparison with previous modularization methods

The performance of the ICSC algorithm was compared with previous approaches for detection of brain functional modules: InfoMap (www.mapequation.org) that minimizes Code Length^23^, Louvain (https://networkx.github.io/) that maximizes Newman’s modularity^22,24^, and FAGSO (https://github.com/CarloNicolini/paco) that maximizes Asymptotical Surprise^10^. To obtain group-level modules from different module detection methods, the consensus clustering for each method was used to obtain ${\boldsymbol{\Pi }}$ by obtaining ${{\bf{s}}}^{k}$ from 2000 iterations for each individual $k$. We reported the group-level modules ${\bf{s}}$ corresponding to the modularization with the optimal value of the fitness function: the modularity ($Q$) for Louvain algorithms, the Code length (${C}_{L}$) for InfoMap, the Asymptotical Surprise (${A}_{S}$) for FAGSO, and the consensus-cost for the ICSC algorithm.

**Figure 4:**
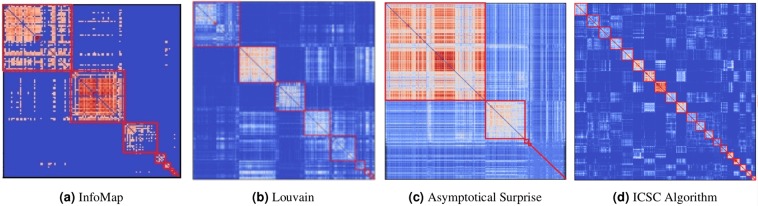
The group consensus matrices obtained for different modular detection approaches. Node indices were reordered to bring nodes in the same module together and the modules are arranged in descending order of their sizes. The red lines highlight the modules in the modularizations for the (**a**) InfoMap (5% top edges, removing isolated nodes), (**b**) Louvain (g = 0:6), (**c**) Asymptotical Surprise, and (**d**) ICSC algorithms.

For InfoMap, we thresholded the connectivity and retained a percentage of weighted edges in the connectivity matrices (from 5% to 20% at steps of 2.5%). On trying different thresholds to the data, we got the best value of InfoMap’s objective function $({C}_{L}=7.185$ for retaining top 5 % edges. We obtained a modularization with 8 modules (containing more than one node) that represent motor merged with auditory system, visual, default mode, fronto-parietal task control and salience systems (Fig. 4(a)). In case of the Louvain algorithm, we varied the $\gamma$ parameter from $0.3$ to $1.5$ (in steps of 0.1)) and obtained $Q=0.358$ for $\gamma =0.6$, with 10 modules for the unthresholded weighted data. We observed that besides the motor, default mode, visual, fronto-parietal task control, and subcortical systems (Figure in Supplementary Materials), the Louvain algorithm was unable to detect other smaller brain functional modules. Even in the group consensus matrix detected using the Louvain algorithm (in Fig. 4(b)), it can be seen that there are smaller clusters that are formed along the diagonal that the algorithm was unable to detect. The FAGSO algorithm found a group-level modularization (Fig. 4(c)) consisting of four modules with more than one node and large number of isolated nodes, that did not provide much information about the brain’s modular structure. We found that the ICSC algorithm was able to detect several functionally relevant, smaller modules that were found to be merged using these algorithms.

Previous studies in the area have used a thresholded and averaged group matrix to detect group-level modules. In order to replicate the previous methods reported in the literature, we performed modularizations on the group-level connectivity matrix obtained by averaging the thesholded individual connectivity matrices. Percolation analysis^61^ was used for thresholding the averaged group matrices. We also used other different thresholds and performed modularization with Louvain, InfoMap and FAGSO. However, similar results were observed as with the matrix obtained from consensus clustering. The results are available in Supplementary Materials.

**Figure 5:**
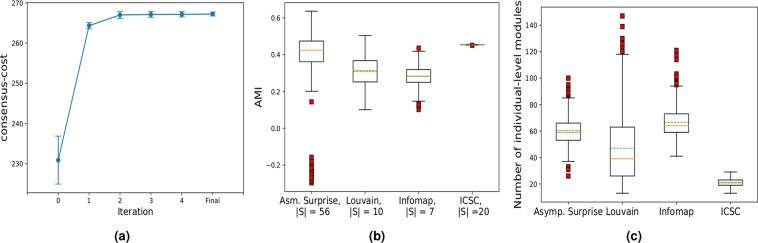
Group-level modularizations for different module detection approaches. (**a**) the cost of ICSC algorithm after different iterations for 100 runs with random initialization, (**b**) the box plot of adjusted mutual information (AMI) between group-level modularizations and individual-level modularizations for the Asymptotical Surprise, Louvain, InfoMap and ICSC algorithms (green dotted line shows the average), and (**c**) the number of individual-level modules for different modularization algorithms.

We calculated the average similarity between individual-level modules and group-level modules obtained from the group consensus matrix with the InfoMap, Louvain, FAGSO and ICSC algorithms (Fig. 5(b)). The most representative ${\bf{s}}$ should have the highest similarity with $\{{{\bf{s}}}^{k}\}$. Group-level modularization from the ICSC algorithm had the highest similarity to the individual-level modularizations. It is also to be noted that the variability in the similarity is the lowest for the ICSC algorithm, further indicating that the group-level modularization obtained from the ICSC algorithm is *consistently* similar to the individual-level modularizations.

We also compared the number of individual-level modules obtained with the best group partition for different modularization algorithms 0(c). The large number of individual-level modules found for FAGSO, InfoMap and Louvain are neuroscientifically implausible and contain a large number of isolated nodes. For ICSC, although we varied the individual-level modules ${{\bf{s}}}^{k}$ in a fixed range, we discovered a varied number of modules for individuals 0(a), which points to variability in individual modular structure.

### Experiments with synthetic data

In order to get quantitative assessment of our methods, we generated synthetic data depicting human brain connectivity. We used the weighted Lancichinetti-Fortunato-Radicchi (LFR) benchmark networks^62,63^ that can mimic properties of resting-state brain networks such as scale-freeness, modules with heterogeneous sizes, and densely interconnected modules.

To generate synthetic networks similar to resting-state brain networks, we studied the properties of the HCP data under consideration. The parameters such as modular sizes and distribution were derived from previously defined functional modules on resting-state fMRI data^2^. Accordingly, synthetic LFR benchmark networks were built with $|\Omega |$ = 264, average degree = 25, maximum degree = 53, minimum degree = 1, degree distribution exponent = 1.13, module size distribution exponent = 1.36, weight distribution exponent = 1.5 and community sizes varying from 5 to 58, proportion of weights inside community ${\mu }_{w}$ = 0.5, and proportion of inter modular edges ${\mu }_{t}$ = 0.5. We obtained the Cholesky Decomposition, ${\boldsymbol{C}}={\boldsymbol{U}}{{\boldsymbol{U}}}^{T}$, of the nearest positive definite matrix from LFR benchmark network ${\boldsymbol{C}}$. We generate ${\boldsymbol{X}}=\{{x}_{i}^{t}{\}}_{i\in \Omega ,0\le t < T}$′ a matrix with $T\mathrm{=1200}$ time points for each region using neuRosim R package and obtain ${\boldsymbol{Y}}={\boldsymbol{UX}}$. We add Rician noise to ${\boldsymbol{Y}}$ and calculate correlation between each region. We varied the signal-to-noise ratio (SNR) by varying the amount of noise added to the network. Since the ground-truth data is available for synthetic data, we used Matthew Correlation Coefficient (MCC) to measure the level of agreement between the detected modules and the ground-truth modules. Given true positives (tp), true negatives (tn), false positives (fp), and false negatives (fn), the Matthew Correlation Coefficient (MCC) is given by

11 $$MCC=\frac{tp\times tn-fp\times fn}{\sqrt{(tp+fp)(tp+fn)(tn+fp)(tn+fn)}}$$

To study the accuracy of group-level modularizations, we generated synthetic brain network datasets representing multiple individuals. Inter-subject differences cause same regions in the brain to participate in different modules across different individuals. To simulate this, we varied the module memberships for nodes that are most likely to be in alternate modules. We identified such nodes by using their betweenness centrality score^64^ that gives the fraction of shortest paths between all nodes passing through a node. We selected a fraction of nodes with high betweenness centrality and varied their modular membership across individuals. To choose an alternate modular assignment for a node, its clustering coefficient was used with a subgraph formed with each module. In addition, we varied the amount of Rician noise added to the network and evaluated the performance of the ICSC algorithm at different signal-to-noise ratios (SNR).

**Figure 6:**
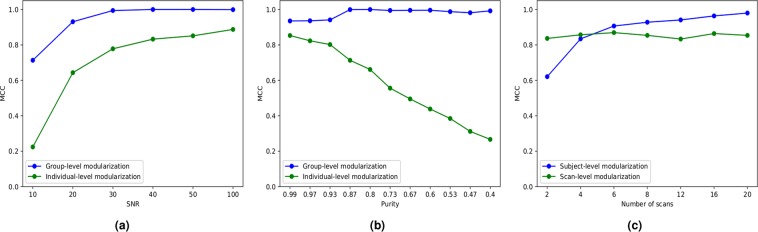
Performances of the ICSC algorithm in deriving group-level and individual-level modules from synthetic data, where (**a**) shows the performance with varying amounts of noise added to the individual scans; (**b**) shows the performance with varying purity for nodes at SNR = 100; and (**c**) shows how the modular detection performance varies with different number of scans.

One hundred runs of the ICSC algorithm were launched on a set of 100 synthetic networks. Initially, a random number of modules were assigned to the individuals in each run and the MCC and the AMI values for the group and individual-level modularizations relative to the ground-truth were noted. We varied the amount of noise introduced in the network and measured the similarity between $\{{{\boldsymbol{s}}}^{k}\}$ and ${\boldsymbol{s}}$ to the ground-truth. We observed that the invividual-level modularizations $\{{{\boldsymbol{s}}}^{k}\}$ improve with decreasing noise, while the group-level modules ${\boldsymbol{s}}$ were close to the ground-truth even for low SNR (SNR $\ge \,30$, Fig. 6(a)). We also observed that the number of group-level modules converged to exactly 14 (number of modules in ground-truth) at the final iteration for all SNRs, indicating the reproducibility of the ICSC algorithm.

To understand the effect of the variability in module assignment to nodes across individuals, we varied the average purity of the nodes. We observed that while the individual-level modularizations $\{{{\boldsymbol{s}}}^{k}\}$ are close to ground-truth at only high purities, the group-level modularizations ${\boldsymbol{s}}$ have a high similarity to the ground-truth throughout (Fig. 6(b)). This shows that the ICSC algorithm is able to detect group-level modularizations reliably irrespective of the purity of nodes in the network. As was the case with noise, it was seen that the ICSC algorithm was able to detect the true number of group-level modules in the data at convergence. Plots for variation of the AMI with different amounts of noise and node modular variations were found to be similar and are attached in supplementary material.

For quantitatively evaluating the reproducibility of the ICSC algorithm in deriving subject-level modules from multiple scans of the same subject, we generated synthetic data for subjects with varying number of scans. We assume that the ROI are involved in the same module in a subject but noise in the connectivity data was varied for different scans. To mimic functional connectivity data for a single subject having multiple scans, we generated 100 LFR benchmark networks (representing different subjects) and generated multiple networks (representing scan sessions) by adding noise to each benchmark network. We generated 2, 4, 6, 8, 12, 16 and 20 networks representing different number of scan sessions for each LFR benchmark network. We report the MCC of the detected modularizations with respect to the ground-truth. We observed that the detection of subject-level modularizations ${{\boldsymbol{s}}}^{k}$ improved as the number of scans per subject increased whereas the scan-level modularizations ${{\boldsymbol{s}}}^{k\sigma }$ had similar performance irrespective of the number of scans (Fig. 6(c)).

## Discussion

We demonstrated the use of ICSC algorithm for detecting brain functional modules on 589 healthy individuals from HCP at the group-level, subject-level, and scan-level. The functional modules were derived from dense and weighted functional connectivity matrices derived from resting-state fMRI scans. At the group-level, we obtained 20 functional modules of heterogeneous module sizes, that have close correspondence to the functional modules discussed in the literature. Not only did we detect previously reported major functional networks, we were able to detect the associated sub-modules in these networks. For example, we were able to differentiate sensory somatomotor network into well-defined modules for feet, hands and mouth; the visual network was also differentiated into three anatomically distinct modules; we were also able to separate the auditory and motor modules that have been found to be clubbed together^48,65^ and we detected a separate module that corresponds to the language network of the brain. The derived group-level modularization by the ICSC algorithm is close to the modularization derived using meta-analyses of functional connectivity data^2^. For quantitative evaluation, we resorted to the use of synthetic networks with known underlying modular structure. To create synthetic data closest to human functional connectivity, we used the LFR networks and generated networks with heterogeneous nodal degree distributions^66^ and modules with heterogeneous sizes^35^. The inter-subject variability was attributed to the modular membership of nodes across individuals and noise. The ICSC algorithm detected the ground-truth modularization with high accuracy and was robust to initialization. We found that the ICSC algorithm was able to detect group-level modularizations close to the ground truth when SNR $\ge \,30$, for different amounts of inter-subject variability, whereas the HCP data has a mean SNR = 728 (minimum SNR = 227.832, SNR = ratio of mean and standard deviation of the time series signal strength).

We have shown that the derivation of group-level modules by ICSC is reproducible since multiple independent runs of the algorithm produced very similar group-level modules. It is to be noted that although the ICSC takes the range of modules $({L}_{min},{L}_{max})$ as user parameters, the upper bound ${L}_{max}$ can be found systematically. We also observed that selecting ${L}_{max}$ beyond a reasonable number (30, in this case) doesn’t change the obtained group-level modularization, but only makes the modularization procedure slower. This makes the ICSC user-friendly since it doesn’t require the user to try out different number of modules or thresholds and does not need to be stopped manually. The derivation of individual-level modularizations is the most expensive step in the algorithm, which can be run parallelly for different subjects, thus making the algorithm fast.

We used the individual-level and group-level modularizations to study the variability in functional architecture across subjects and comparing our results with prior work in the area. The variation in functional membership of brain regions in the frontal, temporal and parietal association cortices is believed to be due to a prolonged period of maturation of white and gray matter^67^, which renders them susceptible to diverse extrinsic individual experiences during a long period of high neuroplasticity^68^. On the other hand, early-developing brain regions that participate in elementary functions like somatomotor/sensory and vision have been found to be stable across individuals^48^. It is also to be noted inter-subject variability in functional connectivity due to aging are well-documented^69,70^, but our participants have a narrow age range (mean 28.33 $\pm$ 3.7) and therefore age-related differences can be disregarded. Upon investigation of the inter-subject variability of functional modules, we uncovered heterogeneous variability in functional membership of regions depending on their functions. We found the subcortical, auditory, dorsal and ventral attentional networks along with inferior brain regions to have high variability across subjects. The attentional networks are located in the association cortices and are therefore highly variable, whereas the variability of basal brain regions can be attributed to the noise in fMRI acquisition due to strong magnetic susceptibility artifacts. Although the high variability in auditory network has not been reported before, but previous studies have reported that auditory network is hard to detect and often fused with a ventral somatomotor network^48,71^. In contrast, the three motor networks for hands, feet and mouth and the visual systems were found to be highly stable across individual-level modularizations. These findings are consistent with the earlier studies that derived inter-subject variability using the intraclass correlation measure^16,29,72^.

Functional modularizations detected at subject-level have wide applications in clinical practice and personalized medicine. However, noise and different cognitive mind states during fMRI scan acquisition introduce variability across fMRI scans of a subject^16,73^. We proposed to use the ICSC algorithm to detect a subject’s modularization from multiple scans. We experiment with the HCP dataset comprising of four scans per subject and with synthetic datasets comprising of different numbers of scans per subject. We observed a statistically significant improvement in similarity between the subject-level and scan-level modularizations at the initial and final iterations of the ICSC algorithm, demonstrating how iterations in the ICSC algorithm improve the subject-level and scan-level modularizations. For the synthetic dataset, we found an increasing agreement between the ground-truth and the subject-level modularization as the number of scans per individual is increased. It is important to note that the initial iteration of the ICSC algorithm corresponds to simple consensus clustering^17^ and our experiments with HCP dataset depict how the multiple modular alignments in the ICSC give more representative subject-level modules from multiple fMRI scans of a subject.

We also performed a comparison of the performance of ICSC with modularization methods used previously. Previous studies have used thresholding^2,10,11,48,65^, averaging^12^, and/or binarizing of functional connectivity matrices for detection of functional modules, which leads to loss of information and introduces artifacts to their results. Although averaging of networks or connectivity matrices has traditionally been performed to compensate for the low SNR of fMRI acquisitions, it ignores the inter-subject and inter-scan variability and leads to spurious and missing functional connections. Present fMRI scans have high SNR due to long acquisition times and lower repetition time so much so that individual-level modularizations have been found to better agree with the individuals’ behavioral characteristics^74^. It is, therefore, necessary to use complete connectivity information of individuals to derive modularizations for functional brain connectivity data. Our results also show that although we do not impose explicit conditions on detection of specific brain functional modules as in^12^, we were successfully able to detect separate modules for the auditory and somatomotor brain systems. Another approach to detect functional modules in the brain is by using time-series of pre-selected seed voxels against the time-series of all other voxels^75^. However, the output of the seed-based approaches are highly dependent on the number and location of initial selected seed regions. In contrast, our approach computes the number of functional modules in a data-driven manner at both individual and group-level and is highly reproducible over independent runs.

The difference in performance between ICSC and previously used algorithms can also be attributed to the quality function being optimized. While, the maximization of ICSC quality function focuses on finding the most representative group-level modularization, other optimization functions find optimized individual-level modularizations. We compared the performance of the ICSC algorithm with several previous used modular detection algorithm, where we derived group-level modularizations with both consensus clustering and averaged subject matrices. For dense unthresholded networks, InfoMap detected modules that correspond to known functional brain systems, but it was unable to identify smaller functional systems in the network. We observed that high density of connections lead to a very few known large sized modules while low thresholds lead to many isolated nodes in the network. For Louvain, we varied the resolution parameter and optimized the modularity of group-level modules. We obtained group-level modules corresponding to known brain functional systems, but failed to resolve several smaller systems in the brain. As described before, this is a consequence of the resolution bias of the quality functions of these algorithms, which depends on the number of connections in the network^76,77^. Although, the normalized cut objective function used in ICSC algorithm also suffers from this resolution bias, we were able to avoid it by specifying a uniform range $({L}_{min},{L}_{max})$ for expected number of modules for the modularization. Finding such a range is not trivial in case of Louvain and InfoMap because it would have to be computed for each individual functional connectivity matrix. Finally, we computed modularizations by using FAGSO algorithm that maximizes Asymptotical Surprise, which has shown to be free from resolution bias^10^. We obtained a group-level modularization containing a few large modules and several small modules composed of a few or isolated nodes.

It should be noted that although weak functional connections have been found to be predictive of intelligence and psychiatric illnesses^42–44^, their effects on modular structure would not be considered due to inability of present module detection methods to work in their presence. The ICSC algorithm considers weak edges in detecting the modular structure and may therefore highlight anomalous functional systems in the brain. Besides showing the neuroscientifically plausibility of the group-level modules computed by ICSC algorithm, we show that the modularization was the closest to the individual-level modularizations, which shows that the ICSC algorithm gives the most representative modular structure.

In the light of the above, the ICSC algorithm becomes a powerful tool to study the brain’s functional architecture during different brain states. The brain states may refer to task states or diseased states. For example, the proposed method has recently been applied to study subject-level brain hubs^55^, where we detected functional modules of the brain and used a novel hub measure, ambivert degree, within the modules to detect biomarkers for Alzheimer’s Disease and Autism Spectrum Disorder.

## Conclusion

We proposed a novel ICSC algorithm to derive a representative modularization for brain functional networks using functional connectivity between different brain regions. Our algorithm takes into consideration multiple dense and weighted functional connectivity matrices and derives a group modularization that best represents the individual modularizations. We derived a group-level modularization for multiple subjects and subject-level modularizations from multiple scans of the same subject. We detected multiple group-level modules having a wide spatial distribution and heterogeneous sizes, corresponding to several known functional systems in the brain. The subject-level modularizations detected by the ICSC algorithm were shown to be representative of the scan-level modularizations. Using group-level modularization we found marked variability in brain modular distribution across individuals, which represents subject differences in functional architecture. On the other hand, subject-level modules gave us a lower inter-scan variability, possibly a result of mind wandering during fMRI scan acquisition. Our methods were demonstrated using resting-state fMRI data from the HCP and synthetic data.

A comparison of the performances of the ICSC algorithm with the InfoMap, Louvain and Asymptotical Surprise shows that the high density connections in the brain functional networks prove detrimental to their brain modularization capability. Further experiments on the synthetic networks with varying amounts of inter-subject variability, noise, and the number of scans demonstrated that the ICSC algorithm is reliably able to detect group-level modularizations and subject-level modularizations. Together, these experiments demonstrate the capability of the ICSC algorithm to identify modules that represent modularizations of multiple subjects or multiple scans of the same subject. This has wide applications in cognitive neuroscience, especially when working at the subject level.

## Supplementary information

Supplementary Information.

Supplementary Information 2.

## Data Availability

The primary data used in this study is from the Human Connectome Project (HCP) of the Washington University-Minnesota Consortium^45,46^ openly available at http://www.humanconnectomeproject.org. The code for the proposed ICSC algorithm is available at https://github.com/SCSE-Biomedical-Computing-Group/ICSC.
