# Supplementary material for: Iterative consensus spectral clustering improves detection of subject and group level brain functional modules
Source: Sci Rep. 2020 May 5;10:7590. doi: 10.1038/s41598-020-63552-0 (PMC7200822; doi:10.1038/s41598-020-63552-0)
Supplement: Supplementary file 1 — Supplementary Information. [file 41598_2020_63552_MOESM1_ESM.pdf]

Supplementary Materials
Iterative consensus spectral clustering improves detection of subject and group level brain functional modules

Sukrit Gupta, Jagath C. Rajapakse

April 22, 2020

Validation on an independent dataset

We used the preprocessed data provided by the Creativity and Affective Neuroscience Lab (led by Dr. Jiang Qiu), Brain Imaging Center of Southwest University. The dataset contained rs-fMRI scans collected from 3T MRI scanners for an 8-min period for 116 cognitively normal subjects from Xinan (First Affiliated Hospital of Chongqing Medical School in Chongqing, China). We ran 100 independent runs of the ICSC algorithm on the validation dataset until convergence. The number of iterations the ICSC procedure took to converge ranged from 2 to 32 (average 9.3 ± 5.5). Out of the 100 independent runs, we selected the run with the maximum consensus-cost and study the individual-level and group-level modularizations for this run. For the validation dataset also the ICSC algorithm detected 20 group-level modules with the sizes of modules ranging from 7 to 19. Although there are differences in the participant demography, scanning protocol and data preprocessing steps we obtained a similar modularization as with the HCP dataset (Fig. S1). We were able to detect sub-modules of the default mode network (Figures S1(b), S1(n) and S1(u)), the motor network (Figures S1(c), S1(i) and S1(j)), the visual network (Figures S1(d), S1(g) and S1(t)), similar to the HCP dataset. We were also able to separate the auditory module from the motor module (Figure S1(l)).

Modules for multiple folds of HCP dataset

In order to understand the reproducibilty of ICSC modularization on a subset of the dataset, we divided the HCP dataset into three folds, such that the average age of the subjects and the gender ratio in each fold is maintained. We ran 20 independent runs on the subject data in each fold and selected the run with the optimal consensus-cost. We compute the adjusted mutual information and the normalized mutual information between the group level modularizations obtained with different folds and with the complete dataset (Fig. S2). We found that the modularizations obtained from different folds were not only very similar to each other (AMI = 0.87, NMI: 0.91), but also to the one obtained with the whole dataset (AMI = 0.92, NMI = 0.94).

Choosing L_{max} for ICSC

The ICSC algorithm expects the user to know the range of expected number of modules given by $(L_{\min}, L_{\max})$. While the lower limit $L_{\min}$ can be fixed based on prior scientific knowledge, the choice of $L_{\max}$ has a trade-off in terms of time and optimization of the quality function associated with it. While smaller than optimal values of $L_{\max}$ will limit the options available for individual-level modularizations generating sub-optimal group-level partitions, larger than optimal values of $L_{\max}$ will lead to more $\boldsymbol{s}^k(l)$ being evaluate and take a longer time to converge.

We demonstrate this by running 10 independent runs with varying $L_{\max} = \{20, 25, 30, 35, 40\}$, and observing the consensus-cost and distribution of number of individual-level modules $\{L^k\}_{k=1}^{K}$. For $L_{\max} = \{20, 25\}$, we found that there is a sharp drop in the $\{L^k\}$ histogram pointing to several individual-level modularizations taking the sub-optimal values for L^k. For $L_{\max} = 30, 35, 40$, we observed a smooth distribution of $\{L^k\}$. The consensus-cost was found to be optimal for $L_{\max} = 30$, with the average number of iterations ranging from 7.0 ± 2.9 for $L_{\max} = 20$ to 32.4 ± 20 for $L_{\max} = 40$.

Intra-subject variability of functional modules

Studies have shown variability in regional functions across multiple scans of the same individual, which is attributable to noise and different states of mind during fMRI acquisition. We measured the variability of the modular memberships of brain regions across multiple scans of the same subject by using the nodal purity. We found a narrow spread of values for nodal purity (average 0.501 ± 0.043) across the subjects. The intra-subject nodal purities corresponding to different brain anatomical locations are shown in figure S4, where the sizes of the nodes correspond to the values

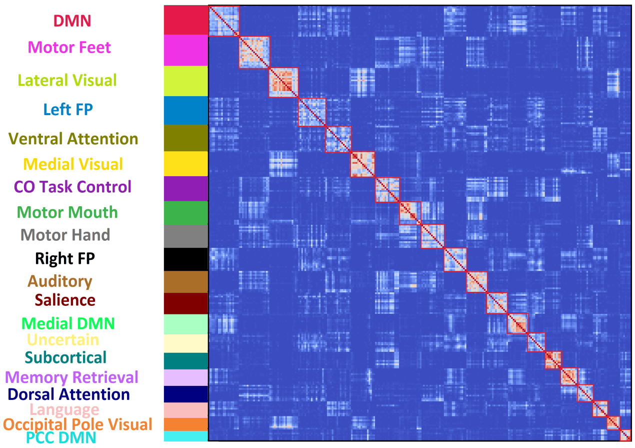

(a)

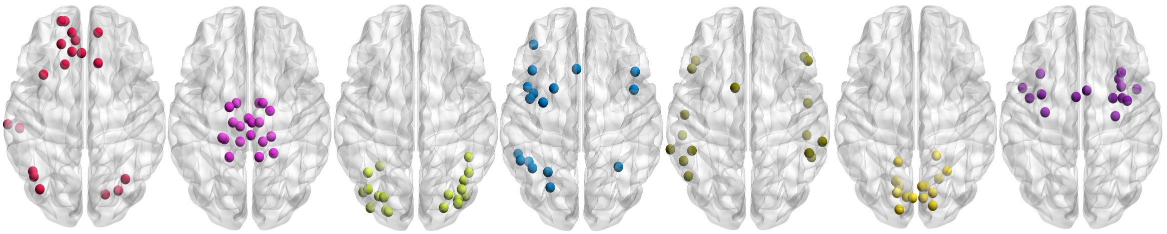

(b) DMN (c) Motor Feet (d) Lateral Visual (e) Left FP (f) Ventral Attn. (g) Medial Visual (h) CO Task Control

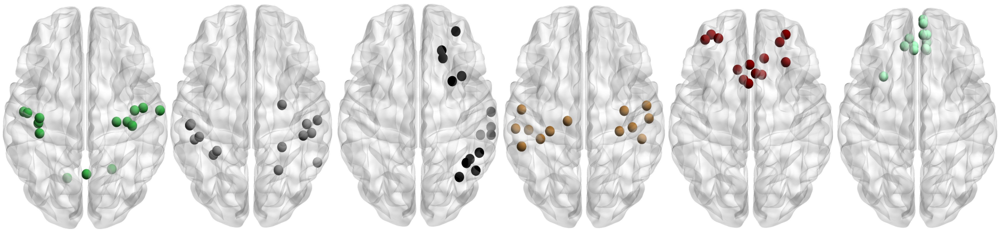

(i) Motor Mouth (j) Motor Hands (k) Right FP (l) Auditory (m) Salience (n) Medial DMN

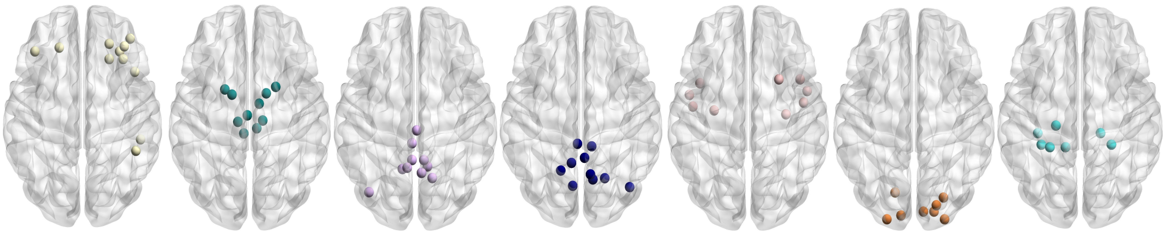

(o) Uncertain (p) Subcortical (q) Memory Retr. (r) Dorsal Attn. (s) Language (t) Occ. Pole Visual (u) PCC DMN

Figure S1: The twenty group-level modules detected by the ICSC algorithm on the resting-state fMRI scans from the validation dataset. (a) the group consensus matrix with reordered node indices to bring nodes in the same module together. (b) to (u) plotted using BrainNetViewer [3] show the ROIs belonging to each module ordered in the descending order of module size. The modules are given names based on functional networks identified by earlier studies or on the anatomical location of constituent regions.

of purity. We found that all the nodes had similar purity in contrast to the inter-subject nodal purity where nodes had pronounced differences in purity. Intra-subject purity scores for different ROIs along with their anatomical coordinates are attached in the file 'Supplement_nodal_purity.csv'.

Using the ICSC algorithm on synthetic data

To quantitatively assess the ICSC algorithm, we generated synthetic data with known ground-truth modular structure mimicking human brain functional connectivity. For evaluating the performance while detecting the group-level

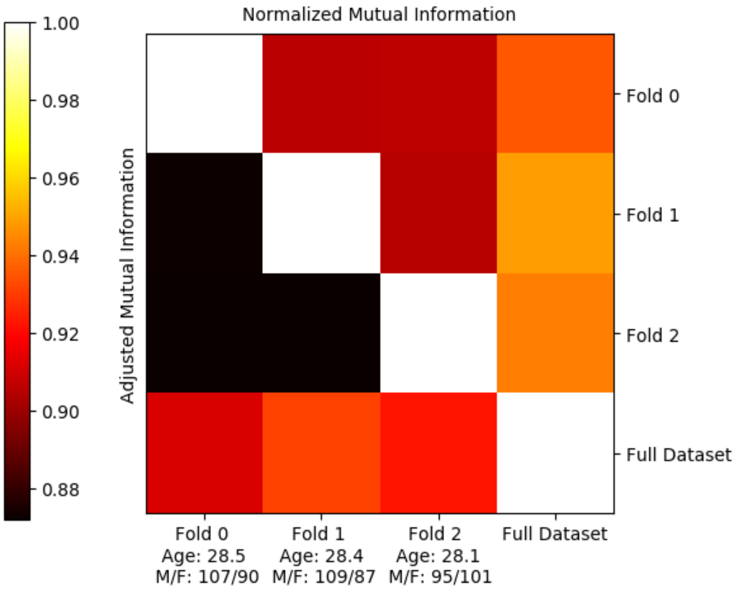

Figure S2: The figure shows the similarity between the group-level modularizations computed for 3 folds of the HCP dataset and the whole HCP dataset. We use two different measures of similarity: the Adjusted Mutual Information (AMI) and the Normalized Mutual Information (NMI).

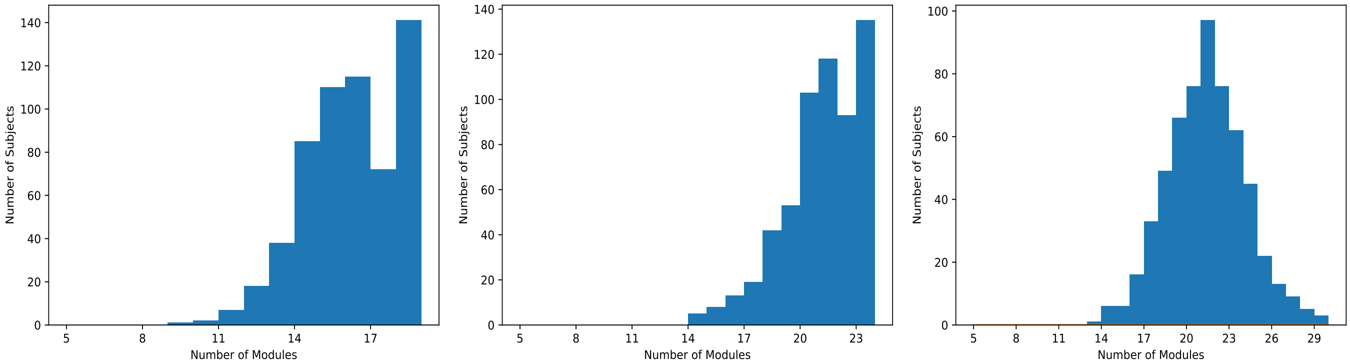

(a) $L_{max} = 20$ (b) $L_{max} = 25$ (c) $L_{max} = 30$

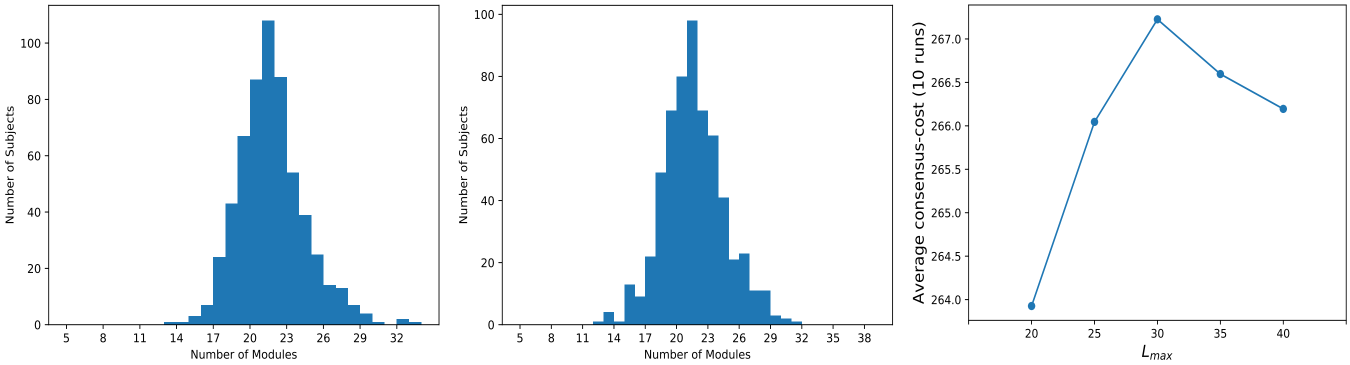

(d) $L_{max} = 35$ (e) $L_{max} = 40$ (f)

Figure S3: Figures (a) to (e) show the distribution of number of individual-level modules $\{L^k\}_{k=1}^K$ for different values of $L_{\max}$. Figure (f) shows the value of the consensus-cost objective function for different values of $L_{\max}$.

modularizations, we generated multiple individual data with varying amount of noise and inter-subject variability in modular assignment. We found that the ICSC algorithm can detect group-level modularizations close to the ground-truth even at low SNR values (Fig. S5(a)) and high inter-subject variability (Fig. S5(b)).

For an individual with multiple scans, we assumed that the functions of brain regions are invariant across the scans

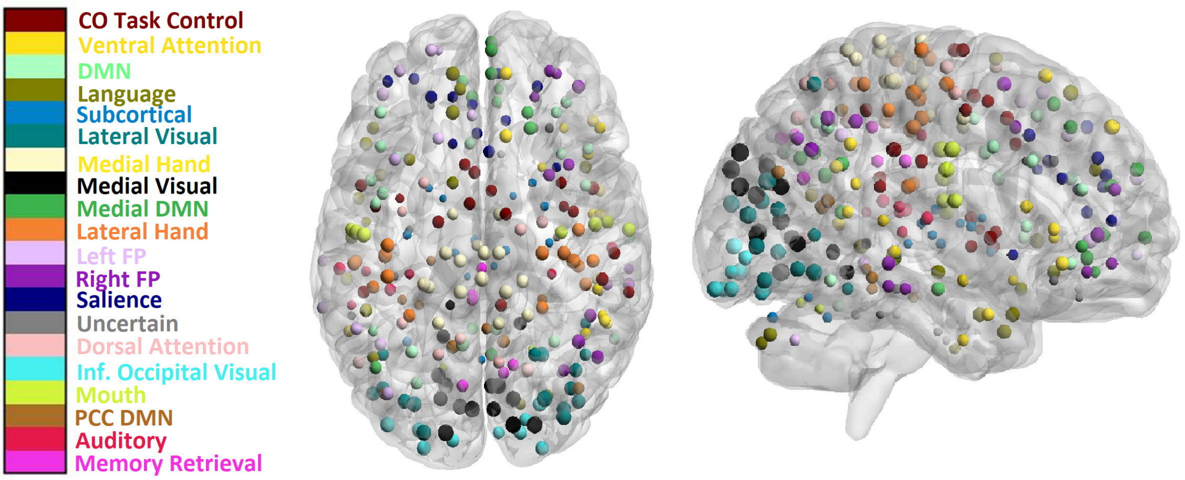

Figure S4: The intra-subject purity corresponding to different anatomical locations of brain ROIs. The sizes of the nodes correspond to the values of purity and the colors denote the functional modules.

and therefore only the number of scans are varied per individual. We found that the individual-level modularization becomes closer to the ground-truth as the number of scans per individual increases (Fig. S5c).

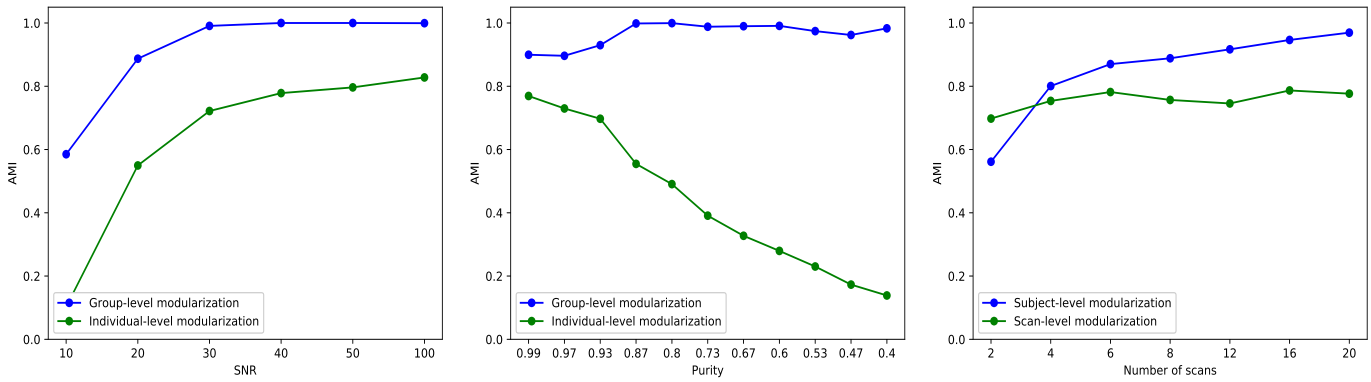

(a) (b) (c)

Figure S5: Performances of the ICSC algorithm in deriving group-level and individual-level modules from synthetic data, where (a) shows the performance with varying amounts of noise added to the individual scans; (b) shows the performance with varying purity for nodes at SNR = 100; and (c) shows how the modular detection performance varies with different number of scans.

Group-level modules from averaged and thresholded connectivity matrices

Previous studies in the area have used a thresholded and averaged group matrix to detect group-level modules. Thresholding removes weak edges and makes the graph sparser, which reduces the computational complexity but loses vital information related to the functional modules. We studied the group-level modularization on the HCP resting state data by using module detection approaches on an averaged and thresholded group-level matrix. For thresholding, we used percolation analysis [1], which iteratively removes weak edges till the connectedness in the network is preserved. Percolation analysis has been applied for thresholding networks for modularization in studies involving humans before [2]. The percolation threshold is where nodes start getting disconnected from the largest component. For our data, this was around 90%. For the sake of completeness, we computed the group-level modules at two other thresholds (10% and 40%) besides the percolation threshold (Fig. S6(a)).

For the Louvain algorithm, we also varied the resolution parameter, γ between 0.3 and 1.3 and chose the modularization with the highest modularity. For low thresholds, we observed isolated nodes in the network. Across different thresholds, we observe that Infomap and Louvain algorithms give a few number of modules, while Asymptotical Surprise gives two large modules (module 1 containing >100 nodes and module 2 containing > 50 nodes) and multiple smaller modules composed of 7 nodes or less.

References

[1] Gallos, L. K., Makse, H. A., & Sigman, M. (2012). A small world of weak ties provides optimal global integration of self-similar modules in functional brain networks. Proceedings of the National Academy of Sciences, 109(8), 2825-2830.

[2] Nicolini, C., Bordier, C., & Bifone, A. (2017). Community detection in weighted brain connectivity networks beyond the resolution limit. Neuroimage, 146, 28-39. [3] Xia, Mingrui, Jinhui Wang, and Yong He. "BrainNet Viewer: a network visualization tool for human brain connectomics." PloS one 8.7 (2013): e68910.

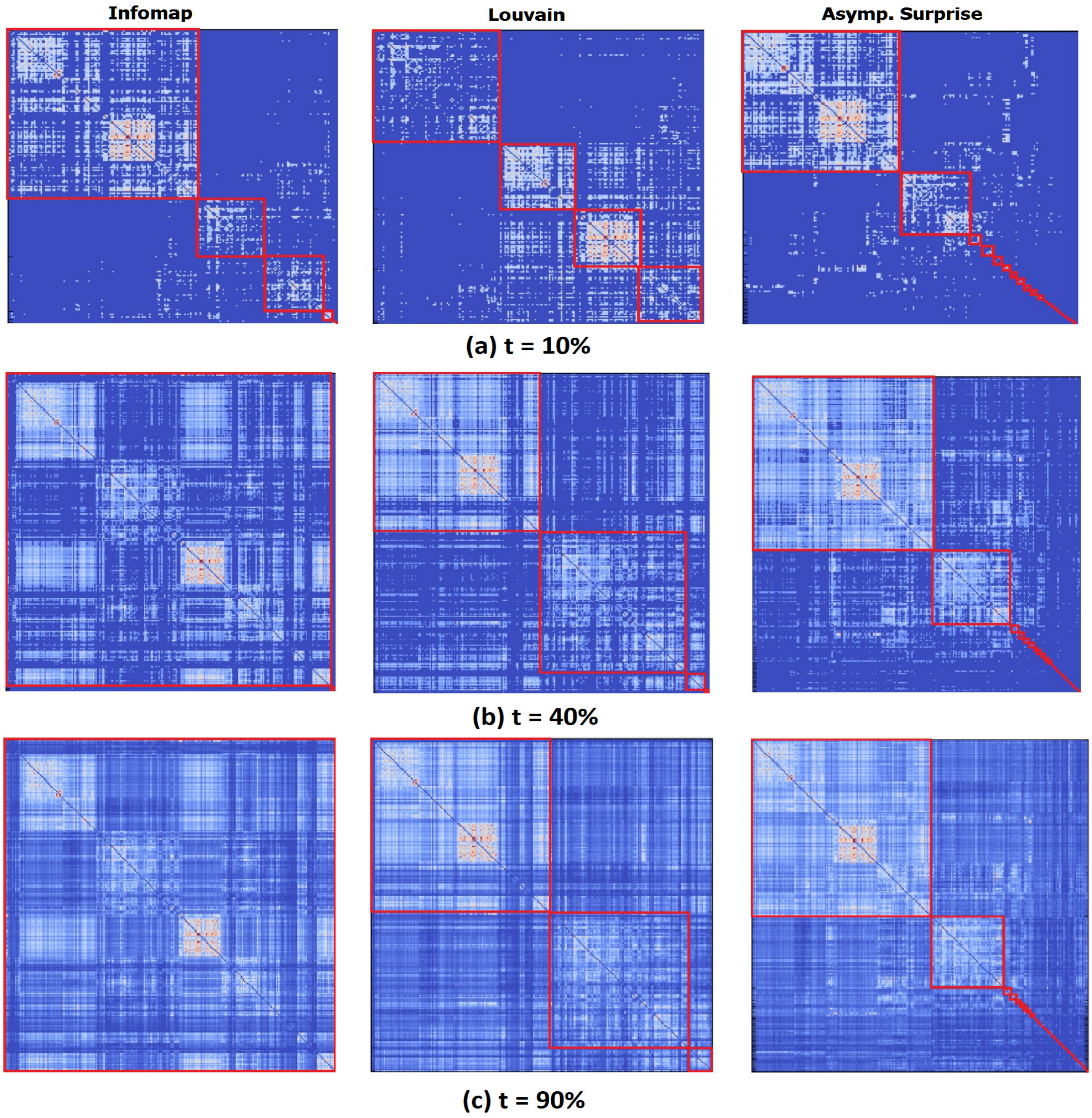

(a)

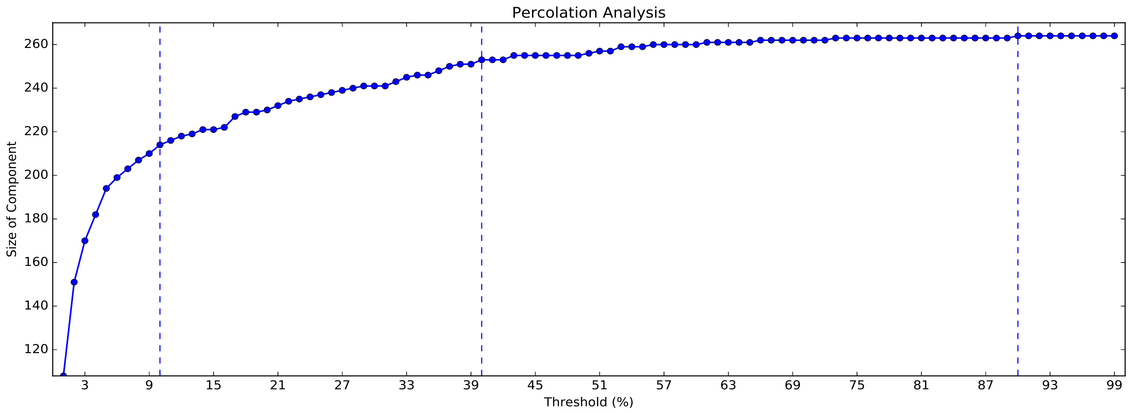

(b) Percolation Analysis

Figure S6: The group-level modular structure obtained with different module detection algorithms at different thresholds of connectivity. The group matrix was obtained from an average of thresholded subject connectivity matrices, where thresholding was performed by percolation analysis. Isolated nodes that result from thresholding are not shown.

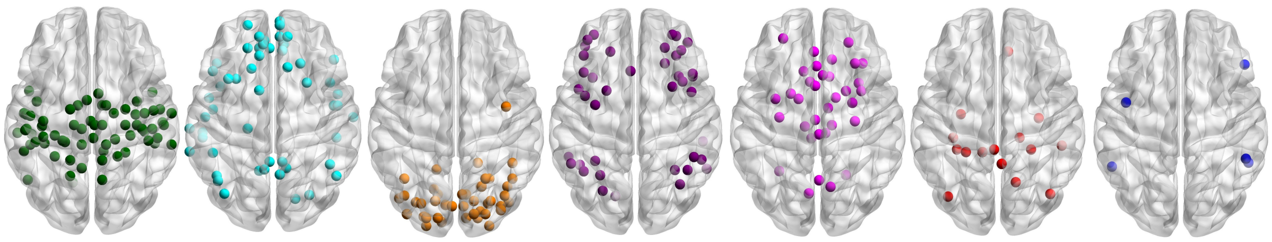

(a) Motor (b) DMN (c) Visual (d) FP (e) Subcortical (f) DMN (g) Ventral Attn.

Figure S7: The 7 group-level modules detected by the Louvain algorithm on the resting-state fMRI scans from the HCP dataset.
